# A sensitive assay for urinary cocaine metabolite benzoylecgonine shows more positive results and longer half‐lives than those using traditional cut‐offs

**DOI:** 10.1002/dta.2153

**Published:** 2017-03-03

**Authors:** Joyce Nickley, Amadeo J. Pesce, Kevin Krock

**Affiliations:** ^1^ Precision Diagnostics LLC, Laboratory 4215 Sorrento Valley Boulevard San Diego CA 92121 USA

**Keywords:** cocaine, urine, benzoylecgonine, sensitive assay, half‐life

## Abstract

Cocaine is a common drug of abuse. To detect its use, a screening detection concentration for the cocaine metabolite benzoylecgonine is commonly set at 150 ng/mL and its confirmatory cut‐off is set at 100 ng/mL. Studies have suggested that these cut‐offs may be set too high, allowing some patients with this substance abuse problem to be missed or improperly monitored. With the advent of liquid chromatography–tandem mass spectrometry (LC–MS/MS) technology it is possible to reliably detect and quantify lower concentrations of its metabolite benzoylecgonine as part of a larger drug panel. One purpose of the study was to establish if there was a significant increase in detection of cocaine use with a ten‐fold more sensitive cut‐off. A very sensitive dilute and shoot assay for benzoylecgonine was developed with a lower limit of quantitation of 5 ng/mL. Validation of the 5 ng/mL cut‐off was achieved by plotting all the positive cocaine observations as a frequency distribution on a logarithmic scale. The number of positive results with measurable concentrations below the typical industry 100 ng/mL cut‐off level but above the high sensitivity 5 ng/mL cut‐off level was observed to be 51.9% of the observed positives. The lower cut‐off also allowed a re‐evaluation of the window of detection after cessation of use. It was observed to be between 17 and 22 days. © 2016 Precision Diagnostics, LLC. *Drug Testing and Analysis* published by John Wiley & Sons, Ltd.

## Introduction

Cocaine is a common drug of abuse. Detecting its use is important in workplace and clinical settings. Cocaine use is primarily detected by testing for its benzoylecgonine metabolite in urine. Typically, the detection is by immunoassay followed by confirmation using a mass spectrometric technique.^1^

Typically, the screening cut‐off detection concentration for the cocaine metabolite, benzoylecgonine, is 150 ng/mL, and its confirmatory cut‐off is set at 100 ng/mL.^1^ Studies have suggested that these cut‐offs may be set too high, allowing some patients who abuse cocaine to be missed.^2^, ^3^ With the advent of liquid chromatography–tandem mass spectrometry (LC–MS/MS) technology, it is possible to reliably detect and quantify lower concentrations of its metabolite benzoylecgonine^4^ as part of a larger drug test panel. From several single‐dose studies, the assumption has been that most of the cocaine is eliminated within a few days of its administration, i.e., the half‐life is on the order of several hours.^5^, ^6^, ^7^, ^8^, ^9^, ^10^, ^11^ In fact, the National Highway Traffic Safety Administration (NHTSA) states that the benzoylecgonine metabolite can be detected up to 10 days after a cocaine binge.^12^ In contrast, other studies have described longer detection times after cocaine use.^10^

The purpose of this study was to establish if there is a significant increase in its urinary elimination using a ten‐fold more sensitive cut‐off.

## Methods

The observations of benzoylecgonine concentrations were obtained from 46 717 pain management and rehabilitation facilities. All patient personal health information was de‐identified and the study was approved by Aspire IRB. In a second study, patients monitored for cocaine relapse were administered multiple urine drug tests over a one‐month period.

For all the studies a very sensitive dilute and shoot assay for benzoylecgonine was developed using a Sciex 6500 instrument with a lower limit of quantitation of 5 ng/mL. To eliminate the possibility of carryover, all concentrations lower than 100 ng/mL were repeated in a separate run with no high benzoylecgonine specimens. Validation of the 5 ng/mL cut‐off was achieved by plotting all the positive cocaine observations as a frequency distribution on a logarithmic scale. The number of positive results with concentrations below the typical industry 100 ng/mL cut‐off level but above the high sensitivity 5 ng/mL cut‐off level were determined. The ratio of these results to the number of low sensitivity positives were used to calculate the false negative rate.

## Results

**Figure 1:**
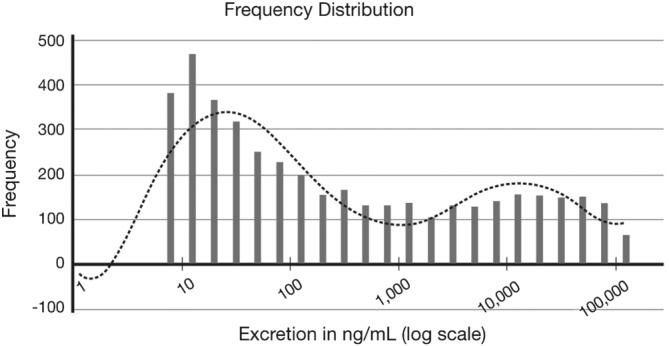
This figure is derived from a previously developed algorithm which plots the log transformed urinary concentrations versus the observed frequency of benzoylecgonine in the study patients.

There were 4252 benzoylecgonine positives above 5 ng/mL. There were 2046 positives above 100 ng/mL. Thus, 2206 would have been called negative by using the 100 ng/mL cut‐off, for a 51.9% false negative rate. Using a previously developed algorithm, we plotted the frequency distribution of benzoylecgonine in these patients (Figure 1). We observed a bimodal distribution of the observed values in Figure 1. This figure clearly shows that more than half of the observed concentrations were below the standard cut‐off of 100 ng/mL.

In a second study, the results of three patients monitored over several weeks are presented in Tables 1, 2, and 3. The patient in Table 1 demonstrated an extended elimination of benzoylecgonine, with detectable levels after 12 days.

**Table 1:** Benzoylecgonine excretion over time for Patient 1

Patient 1	day 0	day 8	day 12
value (ng/ g creatinine)	100	25	16
% difference from previous	—	0.25	0.6
# of half‐lives	—	2	0.8
number of hours between	—	192	96
half‐life (hrs)	—	96	120

**Table 2:** Benzoylecgonine excretion over time for Patient 2

Patient 2	day 0	day 4	day 10	14	25
value (ng/g creatinine)	224	49	19	17	3
% difference from previous	—	0.22	0.39	0.89	0.18
# of half‐lives	—	2.1	1.5	1.1	2.5
number of hours between	—	96	144	96	264
half‐life (hrs)	—	96	96	89	105

**Table 3:** Benzoylecgonine excretion over time for Patient 3

Patient 3	day 0	day 3	day 5	day 10	day 17
value (ng/ g creatinine)	12707	132	46	9	4
% difference from previous	—	0.01	0.35	0.2	0.44
# of half‐lives	—	6.6	1.6	2.4	1.2
number of hours between	—	72	48	120	168
half‐life (hrs)	—	11	30	50	140

The patient in Table 2 demonstrated an extended elimination of benzoylecgonine, with detectable levels after 11 days.

The patient in Table 3 demonstrated an extended elimination of benzoylecgonine, with detectable levels after 17 days. In all three cases, there was no rise in excretion. The pattern was one of slow decline implying there was no reuse. The values range from 11 to 140 h in the same patient indicating that a simple one‐compartment terminal excretion model does not fit the benzoylecgonine excretion pattern.

## Discussion

It is not unexpected that lowering the cut‐off for benzoylecgonine would increase the number of patients positive for cocaine use. Interpretation of such low concentrations can be challenging. We suggest that positive findings in the range of 5 to 100 ng/mL are most likely indicative of less recent cocaine use. We calculated half lives in the tables as estimates of the time for the concentrations of the benzoylecgonine to be one‐half of the previous one. These low concentration findings represent a slow elimination process, which in pharmacokinetic terms is often called beta decay. The length of time for the slow elimination phase of cocaine metabolites has yielded half‐life estimates ranging from 14.6 to 52.4 h.^10^ This means that in the most extreme cases, complete elimination could take two weeks or more. Repeated testing at a one‐week interval should show a decrease in the creatinine corrected concentration of the metabolite. We conclude that values above 100 ng/mL are indicative of recent use.

This work agrees with other studies that have indicated that the 100 ng/mL cut‐off is too high and probably misses those patients engaging in cocaine use.^2^, ^3^

Because of these extended half‐lives compared to the commonly accepted ones we reviewed the scientific literature for confirmation of our observations. We found that these same half‐lives could be calculated from a study by Weiss and Gawin (Table 4). In that study, it was found that heavy cocaine users could have excretion half‐lives of 180 h and benzoylecgonine could be detected up to 21 days after use.^13^

**Table 4:** Benzoylecgonine excretion over time for Patient 4

patient #4	day 0	day 3	day 7	day 10	day 14	day 15	day 21
value (μg/ ml)	34000	15000	800	600	300	500	300
% difference from previous	—	0.044	0.53	0.75	0.5	n/a	0.6
# of half‐lives	—	4.6	1	0.5	1	n/a	0.8
number of hours between	—	72	96	72	96	24	144
half‐life (hrs)	—	15.7	96	144	96	n/a	180

In conclusion, our observations show that the use of lower cut‐offs for benzoylecgonine greatly increases the detection of patients who have used cocaine. In addition, elimination of cocaine is initially very rapid followed by a slower phase taking many days to weeks.

### Disclosures

Authors are employees of Precision Diagnostics.
